# Numerical solution of a diffusion problem by exponentially fitted finite difference methods

**DOI:** 10.1186/2193-1801-3-425

**Published:** 2014-08-11

**Authors:** Raffaele D’Ambrosio, Beatrice Paternoster

**Affiliations:** Via Giovanni Paolo II, 132 - 84084 Fisciano, (Sa), Italy

**Keywords:** Exponentially fitted methods, Partial differential equations, Finite difference methods, Diffusion problems

## Abstract

This paper is focused on the accurate and efficient solution of partial differential differential equations modelling a diffusion problem by means of exponentially fitted finite difference numerical methods. After constructing and analysing special purpose finite differences for the approximation of second order partial derivatives, we employed them in the numerical solution of a diffusion equation with mixed boundary conditions. Numerical experiments reveal that a special purpose integration, both in space and in time, is more accurate and efficient than that gained by employing a general purpose solver.

## Introduction

Many systems of interest in biology and chemistry have successfully been modelled by partial differential equations (PDEs) exhibiting an oscillatory or periodic solution. As an example, we mention oscillatory reaction-diffusion equations, which have periodic waves as fundamental solutions. One particular situation in which the generation of periodic waves has a specific application is intracellular calcium signalling (Atri et al. 1993), described in (Sherratt 1996) and references therein. In this paper, we are particularly aiming to the numerical solution of PDEs with oscillatory solution, by considering in a first analysis the following PDE
1

usually denoted in the literature as *diffusion equation* (compare, for instance, (Hamdi et al. 2007; Isaacson and Keller 1994) and references therein). Such an equation is also called Fourier Second Law when applied to heat transfer; in this case, the function *u*(*x*,*t*) represents the temperature (evolving both in space and in time), while the constant *δ* is the thermal diffusivity of the material. Eq. 1 is also employed, for instance, to model mass diffusion: in this case, it is better known as Fick Second Law, *u*(*x*,*t*) represents the mass concentration and *δ* is the mass diffusivity. We observe that diffusion also plays an important role in magnetic resonance imaging, since it allows to study structural properties of tissues (as in (Ziener et al. 2009), where finite difference methods have been applied to compute numerical solutions).

Classical finite difference numerical methods for PDEs may not be well-suited to follow a prominent periodic or oscillatory behaviour because, in order to accurately follow the oscillations, a very small stepsize would be required with corresponding deterioration of the numerical performances, especially in terms of efficiency. For this reason, many classical numerical methods have been adapted in order to efficiently approach oscillatory problems. One of the possible ways to proceed in this direction is obtained by imposing that a numerical method exactly integrates (within the round-off error) problems whose solution can be expressed as linear combination of functions other than polynomials: this is the spirit of the exponential fitting technique (EF, see (Ixaru and Vanden Berghe 2004; Paternoster 2012) and references therein; also compare (D’Ambrosio et al. 2012a; 2012b; 2011a; D’Ambrosio et al. 2011b; D’Ambrosio et al. 2011c; D’Ambrosio et al. 2013; Ixaru 2012; Vanden Berghe et al. 2003; Vanden Berghe et al. 2001) and references therein for specific aspects of EF-based methods for ordinary differential equations and (Conte et al. 2014; Cardone et al. 2012a, 2012b; Cardone et al. 2010a, 2010b, Ixaru and Paternoster 2001; Kim et al. 2003) for EF numerical integration and its application to integral equations), where the adapted numerical method is developed in order to be exact on problems whose solution is linear combination of the elements of a certain finite dimensional space of functions, usually denoted as fitting space. The specific path we aim to follow in order to numerically solve a diffusion PDE by exponentially fitted methods is essentially the following: we first introduce and analyze exponentially fitted numerical differentiation formulae which approximate the second derivative *∂*^2^*u*/*∂x*^2^, as described in Section “An exponentially fitted second order finite difference”; we next consider diffusion PDEs with mixed boundary conditions and provide a spatial semi-discretization of the problem in Section “A test problem: diffusion equation with mixed boundary conditions”; we finally provide numerical experiments in Section “Numerical results on the semi-discrete model”, where the proposed approach is tested and compared with others known from the existing literature.

## An exponentially fitted second order finite difference

We consider a given function *u*(*x*,*t*) defined on the rectangular domain


and aim to provide a numerical approximation of the second derivative with respect to *x* by the three-point finite difference formula
2

where *h*_*x*_ is a given increment of the *x* variable. The numerical differentiation formula (2) employs, for any given point *x*, its next neighbors *x*−*h*_*x*_, *x*+*h*_*x*_. Other possibilities might be taken into account, e.g. over-next neighbors (as Eq. (25.3.24) in (Abramowitz and Stegun 1964), or Eq. (2.81) in (Gultsch 2004)).

The formula (2) we are going to derive is based on exponential fitting on the fitting space
3

with . To this purpose, following (Ixaru and Vanden Berghe 2004), we introduce the linear operator
4

and, in order to derive the unknown coefficients *a*_0_, *a*_1_ and *a*_2_, we annihilate it on the chosen space (3), i.e.


We observe that each evaluation is always referred to the point (*x*,*t*)=(0,0), due to the invariance in translation of linear operators (compare (Ixaru and Vanden Berghe 2004)). Thus, we obtain the following linear system
5

where *z*=*μh*, in the unknowns *a*_0_, *a*_1_ and *a*_2_, whose solution is
6

Thus, as usual for exponentially fitted formulae, the coefficients are actually functions of *z* = *μh*, hence they are non-constant values and explicitly depend only on the parameter *μ* related to the spatial evolution. The parameter *ω*, which dictates the time oscillations, does not influence the expression of the coefficients of the finite difference and is not directly involved in the spatial discretization. Such a value will next be employed in the time integration of a semi-discrete problem based on (1).

### Order of accuracy

We now analyze the error associated to a generic three-point formula
7

and next specialize the result to the exponentially fitted case considered in the previous section.

#### **Theorem1**

Suppose that *u*∈*C*^4^(*Ω*), where *Ω*=[ *x*−*h*_*x*_,*x*+*h*_*x*_]×[ 0,*T*], being *h*_*x*_>0. If
8

then there exists a constant *C*>0 such that, for any *h*∈(0,*h*_*x*_), we have


#### Proof

In the remainder of the proof, we will use the notation with subscripts to denote partial derivation of the function *u*(*x*,*t*).

We apply the Taylor formula up to the fourth order to the terms *u*(*x*+*h*,*t*) and *u*(*x*−*h*,*t*), obtaining


with *ξ*^+^∈(*x*,*x*+*h*) and *ξ*^−^∈(*x*−*h*,*x*). Hence, by intermediate value theorem, we obtain


with *ξ*∈(*x*−*h*,*x*+*h*). The hypothesis (8) leads to


The thesis holds true with


Roughly speaking, this theorem proves that formula (7) has second order of accuracy provided that its coefficients satisfy (8). This is certainly the case of exponentially fitted formula, depending on the coefficients (6). Thus, we can state the following corollary.

#### **Corollary1**

Suppose that *u*∈*C*^4^(*Ω*), where *Ω*=[ *x*−*h*_*x*_,*x*+*h*_*x*_]×[ 0,*T*], being *h*_*x*_>0. Then, the exponentially fitted finite difference formula (2), whose coefficients are given by (6), has second order of accuracy.

### Trigonometrical case

We now derive a trigonometrically fitted finite difference (2) (which will next be employed in Section “Numerical results on the semi-discrete model”), by annihilating the operator (4) in correspondence of the basis functions


which leads to the linear system
9

whose solution is
10

By similar arguments to those provided in the previous section, we obtain the following result.

#### **Corollary2**.

Suppose that *u*∈*C*^4^(*Ω*), where *Ω*=[ *x*−*h*_*x*_,*x*+*h*_*x*_]×[ 0,*T*], being *h*_*x*_>0. Then, the trigonometrically fitted finite difference formula (2), whose coefficients are given by (10), has second order of accuracy.

### Recovering the classical first order finite difference

We finally recover the classical first order finite difference for the numerical approximation of *u*_*xx*_, by annihilating the evaluations of the linear operator (4) on the monomial basis {1,*x*,*x*^2^}, i.e.


This leads to the following linear system of equations
11

whose solution is
12

and leads to the well-known classical finite difference
13

which is known to have second order of accuracy. We observe that the coefficients (6) of the finite difference (2), when *z* tends to 0, tend to the classical coefficients (12): this confirms that the exponentially fitted finite difference has second order of accuracy. Analogously, we also recover the second order of accuracy of the trigonometrically fitted finite difference (2), with coefficients (10).

## A test problem: diffusion equation with mixed boundary conditions

We now consider the following diffusion problem with mixed boundary conditions (Hamdi et al. 2007; Schiesser 1991; Schiesser and Griffiths 2009)
14151617

in the rectangular domain [ *x*_0_,*X*]×[ *t*_0_,*T*]. We aim to solve this problem by exponentially fitted methods taking into account the behaviour of the solution in time and space, by suitably applying the method of lines (compare (Isaacson and Keller 1994; Schiesser 1991; Schiesser and Griffiths 2009) and references therein). Hence, we now present the semi-discretized problem with respect to the spatial variable.

### Spatial semi-discretization of the operator

As announced, we now aim to provide a spatial semi-discretization of the operator. Thus, we consider *N* equidistant points in the spatial interval [ *x*_0_,*X*] and denote by *h*_*x*_ the distance between two consecutive points. The semi-discretized domain, denoted by *D*_*h*_, results to be


We next denote by *u*_*j*_(*t*)=*u*(*x*_*j*_,*t*), 0≤*j*≤*N*−1. As a consequence, the original problem (14)-(17) is transformed in the following system of *N* first order ordinary differential equations
181920

with initial values *u*_*j*_(*t*_0_)=*u*_0_(*x*_*j*_), 0≤*j*≤*N*−1. We observe (compare (Hamdi et al. 2007) and references therein) that Eq. 18 arises from the boundary condition (16), while (20) is obtained by (17), by observing that


which implies that *u*_*N*_(*t*)=*u*_*N*−2_(*t*). Eq. 19 is simply obtained through a replacement of the second derivative with the finite difference. Of course, the nature of the semi-discretization strongly depends on the type of chosen finite difference (e.g. trigonometrical, exponential or classical).

## Numerical results on the semi-discrete model

**Figure 1:**
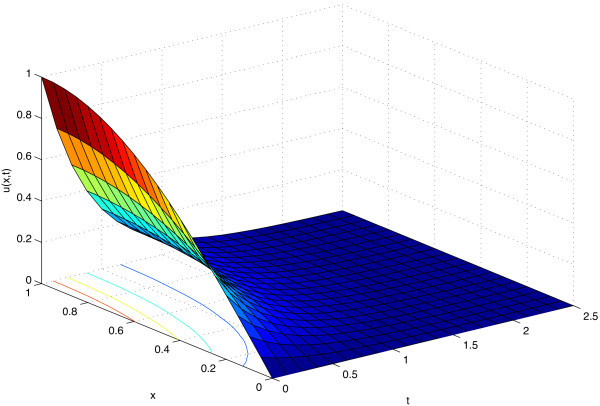
**Profile of the solution of problem (**14**)-(**17**), in the rectangular domain [ 0,1]×[ 0,2** ***.*** **5].**

We now aim to solve problem (14)-(17) with *δ*=1 and


whose exact solution is

 represented in Figure 1.

The solution is thus oscillatory in the space variable and exhibits an exponential decay with respect to the time variable. Due to this qualitative behaviour, we proceed as follows:

- we consider the semi-discrete problem (18)-(20) obtained by discretizing the spatial derivative with both the classical finite difference (i.e. *a*_0_, *a*_1_ and *a*_2_ given by (12)) and the trigonometrically fitted one (i.e. *a*_0_, *a*_1_ and *a*_2_ given by (10));
- we perform a time integration for both the semi-discretized problems, i.e. those obtained by approximating the second derivative with the classical finite difference and the trigonometrical finite difference. For each problem we consider both classical constant coefficient numerical methods (i.e. those implemented in the Matlab ode15s routine) and by exponentially fitted methods (i.e. the EF-based explicit Runge-Kutta method provided in (Vanden Berghe et al. 1999) and the EF-based Lobatto IIIA method introduced in (Vanden Berghe et al. 2003)).

It is worth observing that the application of numerical methods depending on non-constant coefficients is strongly connected to the knowledge of a good approximation of the involved parameters (compare (D’Ambrosio et al. 2012a; 2012b; Ixaru et al. 2002; Vanden Berghe et al. 2001) and references therein). In our test example, as a preliminary analysis, we a-priori know the exact values of the parameters, i.e. the frequency of the spatial oscillations and the parameter dictating the exponential decay in time, and exploit them in the integration, as often happens in the exponential fitting approach.

**Figure 2:**
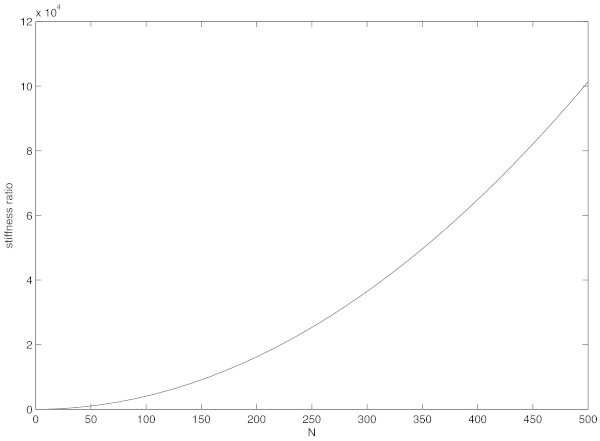
**Stiffness ratio of the semi-discretized problem (**19**)-(**20**), with** ***a***_**0**_ **,** ***a***_**1**_ **,** ***a***_**2**_ **given by (**12**).**

Through the results reported in Tables 1 and 2, we can observe what follows:

- the application of an explicit time integrator leads to an unstable behaviour. This fact is not surprising, because the semi-discretized problem results to be stiff. In order to better understand this aspect, we look at the semi-discrete problem (19)-(20) (Eq. 18 is neglected because it is actually an independent quadrature problem), which can be written in matrix form as
  with
- The stiffness ratio associated to the above system of ordinary differential equations is depicted in Figure 2 for the classical semi-discretization, i.e. *a*_0_, *a*_1_, *a*_2_ are given by (12). Similar values of stiffness ratio are obtained also in the exponential and trigonometrical cases, which are here omitted for brevity. One can easily recognize that the more *N* is large, the more the problem is stiff, thus it makes nonsense to solve it by explicit numerical methods;
- the more the numerical method is adapted to the nature of the solution, the more the result is accurate: in fact, the exponentially fitted time-integration via the adapted Lobatto IIIA method (derived in (Vanden Berghe et al. 2003)) is more accurate than the Matlab ode15s routine, when both are applied to the spatially semi-discrete problem. This is due to the fact that the solution exhibits an exponential behaviour with respect to the time variable, thus the exponentially fitted Lobatto IIIA method is more adapated to the problem, with evident advantages in the accuracy of the numerical solution. The most accurate combination is that given by the trigonometrically fitted finite difference and the exponentially fitted Lobatto IIIA method: indeed, in this way, the numerical procedure is strongly adapted to the behaviour of the solution, which is trigonometrical with respect to the spatial variable and exponential with respect to time.

**Table 1:** **Norms of the global errors arisen in the application of different spatial finite differences and time solvers to the semi-discrete model (**18**)-(**20**) with** ***N*** **=21, in the rectangular domain [0,1]×[0,2.5]**

Time solver	Classical finite difference	Trigonometrical
		finite difference
ode15s	3.17e-3	3.12e-10
EF-based explicit	unstable	unstable
RK method		
EF-based Lobatto	3.22e-3	4.11e-12
IIIA method

**Table 2:** **Norms of the global errors arisen in the application of different spatial finite differences and time solvers to the semi-discrete model (**18**)-(**20**) with** ***N*** **=41, in the rectangular domain [0,1]×[0,2.5]**

Time solver	Classical finite difference	Trigonometrical
		finite difference
ode15s	7.93e-4	3.09e-10
EF-based explicit	unstable	unstable
RK method		
EF-based Lobatto	7.96e-4	2.26e-12
IIIA method		

## Conclusions

We have presented an alternative approach to numerically solve partial diffential equations. This approach is based on the exponential fitting technique, which consists in specializing a numerical method to the behaviour of the solution. In our initial analysis, we have considered a diffusion problem with mixed boundary conditions, semi-discretized according to different finite differences approximating the spatial derivative, and solved by employing both general and special purpose numerical methods. In applying special purpose methods, we have supposed that the values of the parameters are a-priori known: in further developments of this research, we will remove this hypothesis, and consider suitable procedures to accurately derive an approximation of the unknown parameters, following the lines drawn in (D’Ambrosio et al. 2012a; 2012b; Ixaru et al. 2002; Vanden Berghe et al. 2001). As highlighted by the numerical evidence, the more the numerical method is adapted to the nature of the solution, the more the result is accurate. The achieved results make us hope that such an approach might be successfully employed to many other partial differential equations, which represent our future perspective of prosecution along the path drawn in this paper.
